# Silencing of Soybean Raffinose Synthase Gene Reduced Raffinose Family Oligosaccharides and Increased True Metabolizable Energy of Poultry Feed

**DOI:** 10.3389/fpls.2017.00692

**Published:** 2017-05-16

**Authors:** Michelle F. Valentine, Joann R. De Tar, Muruganantham Mookkan, Jeffre D. Firman, Zhanyuan J. Zhang

**Affiliations:** ^1^Plant Transformation Core Facility, Division of Plant Sciences, University of MissouriColumbia, MO, USA; ^2^Division of Animal Sciences, University of MissouriColumbia, MO, USA

**Keywords:** *Glycine max*, RNAi, raffinose, oligosaccharides, true metabolizable energy

## Abstract

Soybean [*Glycine max* (L.) Merr.] is the number one oil and protein crop in the United States, but the seed contains several anti-nutritional factors that are toxic to both humans and livestock. RNA interference technology has become an increasingly popular technique in gene silencing because it allows for both temporal and spatial targeting of specific genes. The objective of this research is to use RNA-mediated gene silencing to down-regulate the soybean gene *raffinose synthase 2* (*RS2*), to reduce total raffinose content in mature seed. Raffinose is a trisaccharide that is indigestible to humans and monogastric animals, and as monogastric animals are the largest consumers of soy products, reducing raffinose would improve the nutritional quality of soybean. An RNAi construct targeting *RS2* was designed, cloned, and transformed to the soybean genome via *Agrobacterium-mediated* transformation. Resulting plants were analyzed for the presence and number of copies of the transgene by PCR and Southern blot. The efficiency of mRNA silencing was confirmed by real-time quantitative PCR. Total raffinose content was determined by HPLC analysis. Transgenic plant lines were recovered that exhibited dramatically reduced levels of raffinose in mature seed, and these lines were further analyzed for other phenotypes such as development and yield. Additionally, a precision-fed rooster assay was conducted to measure the true metabolizable energy (TME) in full-fat soybean meal made from the wild-type or transgenic low-raffinose soybean lines. Transgenic low-raffinose soy had a measured TME of 2,703 kcal/kg, an increase as compared with 2,411 kcal/kg for wild-type. As low digestible energy is a major limiting factor in the percent of soybean meal that can be used in poultry diets, these results may substantiate the use of higher concentrations of low-raffinose, full-fat soy in formulated livestock diets.

## Introduction

Soybeans [*Glycine max* (L.) Merr.] are the number one protein source for animal feed in the world, accounting for 69% of global protein consumption with poultry and swine—both monogastric animals—being the major consumers (Cromwell, 2012). However, soy's use in monogastric animal diets must be supplemented with alternate sources of carbohydrates such as corn or other cereal grains to compensate for the overall low level of digestible carbohydrates. One of the major limitations of soy carbohydrates is the presence of the indigestible raffinose family oligosaccharides (RFOs): raffinose, stachyose, and verbascose. These compounds are derived from sucrose, which has a positive effect on metabolizable energy, but due to their α-1,6-glycosidic bond, monogastric animals are unable to digest RFOs. Oligosaccharides pass undigested through the upper gut of the animal, and are then fermented by anaerobic microbes in the lower gut. This fermentation produces carbon dioxide, methane, and hydrogen, causing flatulence and digestive disturbance in the animal. Further, it has been shown that presence of RFOs in animal diets caused the feed to pass quicker through the digestive system, reducing the amount of other nutrients absorbed from the feed (Coon et al., 1990).

In plants, raffinose and related compounds are believed to provide protection from various stresses such as tolerance to drought (Wang et al., 2009), seed desiccation (Koster and Leopold, 1988), and cold (Zuther et al., 2004), and the scavenging of reactive oxygen species (Nishizawa et al., 2008) and partitioning of carbohydrates during times of stress (ElSayed et al., 2014). In soybean, slow drying of immature seeds increases RFO accumulation, and a positive correlation between seed stachyose content and desiccation tolerance has been established (Blackman et al., 1992). RFOs may also be a readily accessible energy source for germinating seeds, as inhibiting RFO metabolism drastically decreases germination of pea seeds (Blöchl et al., 2007). However, raffinose and stachyose metabolism is not required for soybean seed germination, as demonstrated in lines bred for low seed RFO content (Dierking and Bilyeu, 2009b). It is hypothesized that the primary functions of RFOs are storage and transport, and although high accumulations of RFOs during times of stress do provide protection, stress protection is not their exclusive role in plants (Sengupta et al., 2015).

Raffinose biosynthesis in developing soybean seeds is catalyzed by raffinose synthase 2 (RS2), which is encoded by Glyma06g18890 (Dierking and Bilyeu, 2008). RS2 catalyzes the reaction: sucrose + galactinol 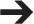 raffinose + myo-inositol. The subsequent conversions of raffinose to stachyose, and stachyose to verbascose produce the spectrum of raffinose family oligosaccharides, though verbascose content in soybean seeds is negligible (Kumar et al., 2010). RS2 is considered the committed step in RFO biosynthesis, and, as a result, down-regulation of this gene should lead to increased sucrose, and decreased raffinose and stachyose. Successful soybean breeding efforts to reduce raffinose content in mature seeds have lowered raffinose content from about 1–1.5% in wild-type (WT) to nearly undetectable levels in mutant lines. Two major mutations in the soybean gene *RS2* have been identified and associated with the low-raffinose phenotype (Kerr and Sebastian, 2000; Dierking and Bilyeu, 2009a; Bilyeu and Wiebold, 2016). The two mutants showed relatively variable low raffinose phenotypes across environments. The absolute quantities of sucrose, raffinose, and stachyose are highly dependent on location and planting date. Agronomic traits such as emergence, yield, maturity, seed protein, and oil content were not affected in one mutant (W331) (Neus et al., 2005).

Although two individual mutant lines with a low-raffinose phenotype have been identified, additional association of the RS2 gene with altered carbohydrate phenotype could provide separate confirmation of the gene-to-phenotype association and low RFO soybean line of commercial value. RNAi-mediated transgenic knockdown of gene expression is a unique and efficient tool to analyze the contribution of a gene to a particular phenotype (Hannon, 2002). Benefits of RNAi technology include sequence specificity, the ability to modulate gene expression in a tissue- and time-dependent manner, and silencing of multiple genes in parallel. RNAi can also result in varying levels of partial knockdown, avoiding lethality. Further, RNAi phenotypes are dominant, so the desired phenotype can be observed in the first generation after transformation, rather than requiring the extra breeding effort to produce homozygous knockouts needed for mutagenesis (Small, 2007).

To date, no successful RNAi of the soybean RS2 gene has been reported. This research represents the first successful transgenic alteration of soybean raffinose content, leading to increased true metabolizable energy of poultry feed.

## Materials and methods

### Vector construction and soybean transformation

To construct binary construct pMU2T-bar-RS2 we first built a 2 T-DNA binary vector for the cloning of expression cassettes by amplifying T-DNA borders using PCR and clone them into the binary vector pPZP201 (Hajdukiewicz et al., 1994). To do this two PCR reactions were done to amplify the borders using two sets of primer pairs (Table S1), and the same template was amplified for one left border. After amplification, both PCR products were cloned into pGEM-T Easy (Promega, Madison, USA) and two right borders from pGEM-T Easy were digested using *Pst*I and *Sph*I, and ligated into *Pst*I and *Sph*I sites of pPZP201. Afterwards the left border in pGEM-T Easy was cut with *Aat*II and *Sph*I, and further cloned into the vector with the two right borders. This two T-DNA vector resulted in pMU-2T. The RNAi expression cassette digested from pMU103 (Flores et al., 2008) and then cloned into the first T-DNA region in pMU-2T. Then, a *bar* expression cassette was PCR-amplified from pZY102 (Zeng et al., 2004) as *Eco*RI-*Pst*I (blunt-ended) fragment and cloned into *Eco*RI/*Sac*I (blunt-ended) sites of the second T-DNA region in a reverse orientation. *Agrobacterium*-mediated soybean transformation followed our improved protocol derived from previous one (Zeng et al., 2004; Flores et al., 2008) using an elite soybean genotype “Maverick.”

### PCR confirmation of transgenes

Primers were designed to flank from the rice waxy gene intron to OCS terminator for detection of the gene of interest, and within the *bar* gene for detection of the selectable marker gene (Table S1). Leaf samples were collected from plants in greenhouse. Genomic DNA was extracted using an SDS/EDTA buffer, and PCR conditions were: 95°C hot start 30 s; followed by 35 cycles of 95°C denaturing 10 s, 55° or 56°C annealing 10 s, and 72°C extension 1 min; then 72°C final extension 10 min. PCR products were analyzed on agarose gel and events were considered transgenic if they displayed a 500 bp band for the gene of interest and an 800 bp band for the *bar* gene.

### Progeny segregation analysis

To determine the segregation of gene of interest and selectable marker gene, at least 20 plants from each T0 event were screened using leaf-paint analysis using 100 mg/L glufosinate and PCR. The leaf-paint and gene of interest PCR were carried out as described for the T0 generation, but new PCR primers were designed for the *bar* gene to flank from the CaMV35S promoter to the *bar* coding sequence to prevent false positive amplification of *bar*. T2 progeny from the T1 generation were similarly analyzed to identify homozygous T1 lines for subsequent study.

### qRT-PCR of mid-mature seeds

Mid-mature seeds were analyzed by qRT-PCR to determine the relative expression of endogenous *RS2* mRNA in WT “Maverick” genotype as well as transgenic seeds (Dierking and Bilyeu, 2008). Nine seeds were collected from each plant and ground with liquid nitrogen. RNA was extracted with Trizol reagent, purified with phenol:chloroform:isoamyl-alcohol (24:1:1 v/v) and resuspended overnight in 50 μL of ddH_2_O at 4°C. The DNA was then removed by DNaseI treatment (Promega) and RNA was stored at –80°C. For T1 seed analysis, about 20 seeds from each plant were first seed-chipped by subjecting about 1/6 of the seed to DNA extraction and PCR analysis as described above. Total RNA was then extracted from the rest of the seed whose chip was PCR-positive for RS2 transgene. For greenhouse T3 seeds, three mid-mature seeds were sampled from each of the top, middle, and bottom of the plant canopy, pooled and flash-frozen in liquid nitrogen, then stored at −80°C before RNA extraction. For field T3 seeds, nine mid-mature seeds were collected from each of 10 plants of each WT and RS2 transgenic. To account for variability across the field plot, plants were sampled randomly across the plot space and the seeds for each genotype were pooled. A subsample of 10 seeds was used for each of three RNA extractions for each genotype. Total RNA was extracted and analyzed by qRT-PCR as done in T1 seeds.

For qRT-PCR reaction, random hexamers were annealed to DNase-treated cDNA for reverse transcription by M-MLV RT (Promega) at 37°C for 1 h. Then cDNA was diluted 1:3 with ddH_2_O and stored at −20°C. One microliter of diluted cDNA was used for qPCR in a BIO-RAD CFX96 Real Time System machine. qRT-PCR was performed with 5 μL SsoAdvanced Universal SYBR Green Supermix (BioRad) in a 10 μL total reaction volume. Forward and reverse primer sequences for endogenous *RS2* are from Dierking and Bilyeu (2008). Elongation factor 1α was used as an internal control. Three technical replicates were used for each of three biological replicates for each study. The qPCR reaction conditions were: 95°C hot start 3 min, followed by 40 cycles of: 95°C denature 10 s and 55° or 56°C annealing 30 s, 72°C extension 1 min and 72°C final extension 10 min, then a melt curve was analyzed from 65° to 95°C with measurements taken every 0.5°. Each cDNA sample was triplicated on the 96-well-plate to measure technical variability. A no-RT sample was included to confirm the lack of genomic DNA contamination.

### Southern blot

Leaf painting and PCR were first used to screen T1 plants representing RS2 transgene integration without the *bar* gene using primer pairs expanding from OCS 3′ region to the 5′ region of rice waxy A gene intron (Table S1). Genomic DNA (gDNA) was extracted from young fully-expanded leaves of T1 young seedlings representing each primary (T0) event with CTAB buffer, purified with 24:1 (v/v) chloroform:isoamyl alcohol, resuspended in TE buffer, and treated with RNase. Thirty micrograms of gDNA was digested with *Nco*I in 300 μL total volume, ethanol precipitated and was run in 1% agarose gel and blotted onto Amersham Hybond-XL membrane (GE Life Sciences) which was hybridized by ^32^P-labeled dATP probe prepared using Prime-It II Random Primer Labeling Kit (Agilent Technologies). The membrane was washed and exposed onto X-ray film before analysis.

### HPLC analysis of carbohydrate profiles

Five seeds from each T1 line were lyophilized, pooled, and ground with liquid nitrogen and analyzed following the protocol of Dierking and Bilyeu (2009a). Results for each oligosaccharide were given as percent of total carbohydrate fraction by weight. Sucrose was evaluated using the Megazyme Sucrose/D-Glucose Assay Kit (Megazyme Co, Ireland) which uses a glucose oxidase and peroxidase method to detect sucrose and glucose content in foodstuffs. This assay can accurately measure seed sucrose content (Hou et al., 2009). For T3 generation, 15 seeds from field-grown transgenic (MM3-2-15) and WT (Maverick) were pooled, ground, extracted, and HPLC-analyzed as before. Each extraction sample was run on the HPLC machine twice. Two replicates of the Megazyme assay were also run to determine sucrose content.

### Field increase of transgenic seed

Seeds from each of RS2 transgenic (MM3-2-15) and WT were planted and cared under the field conditions. Emergence rate was recorded 13 days after seed planting. All transgenic plants were within 20-foot border zones surrounding the plot. Plants were grown to physiological maturity and dry seeds were harvested manually.

### Seed composition analysis

To assess if alterations in carbohydrate content affected protein, oil, or fiber composition, ground seed was analyzed by MU Agriculture Experiment Station Chemical Laboratory for analysis. Amino acid compositions were analyzed using AOAC Official Method 982.30, crude protein was measured by the Kjeldahl method, AOAC Official Method 984.13, crude fiber was measured by AOAC Official Method 978.10, crude fat was measured by Ether Extraction, AOAC Official Method 920.39, ash was measured by AOAC Official Method 942.05, and moisture was measured by AOAC Official Method 934.01, 2006.

### Soybean meal preparation for animal feeding study

Whole soybean seeds were roasted at 55°C for 1 h and allowed to cool, then ground in a Wiley laboratory mill fitted with a 3 mm screen 1 day prior to feeding. The ground soybean was weighed into 30 g portions and transported to the poultry research farm for feeding.

### Precision-feeding assay

Chickens were housed in standard experimental individual feeding cages. Fourteen-week-old Single Comb White Leghorn roosters were obtained from a local pullet farm, and at around 18 weeks of age, cecectomy surgeries were performed following the procedures described in Parsons (1985). After 4 weeks of recovery, the precision feeding assay was conducted using 14 total roosters. Roosters were weighed and separated into two groups with each group having approximately equal total chicken weight. Roosters were fasted for 24 h to empty the digestive tract and then tube-fed 30 g each of WT or low-raffinose soybean. Excreta was collected every 4 h for the first 24 h, and then again at the 48 h mark. A second experiment was conducted without feeding to measure the endogenous excreta loss during a 48 h period following 24 h of fast.

### Sample preparation and analysis

After drying the excreta samples for 24 h at 65°C, weights were recorded and then all samples from each chicken were pooled. Dried excreta were ground with a 1 mm screen and gross energy was measured by a Parr model 1,341 oxygen bomb calorimeter and model 1,108 oxygen combustion vessel calibrated with benzoic acid (Parr Instrument Company, Moline, IL). Dry matter digestibility and true metabolizable energy were calculated as in Sibbald (1976), i.e.,

$$\text{TME}(\text{kcal/kg})=\frac{\left({\text{GE}}_{\text{f}}\times {\text{F}}_{\text{i}}\right)-\left(\text{GEe}\times ({\text{Y}}_{\text{f}}{\text{-Y}}_{\text{e}})\right)}{{\text{F}}_{\text{i}}}$$

Where,   GE_e_ = gross energy of the excreta (kcal/kg)

GE_f_ = gross energy of the feed (kcal/kg)
F_i_ = feed input (g)
Y_f_ = excreta from fed chicken (g)
Y_e_ = excreta from unfed chicken (g).

### Research compliance

Our animal feeding test followed the Animal Care and Use protocol #8477, approved by the University of Missouri Institutional Animal Care and Use Committee.

### Statistical analysis

SAS software (Version 9.4) was used for all data analysis. Means were separated by PROC ANOVA and were considered significant at *p* = 0.05, followed by different letters where appropriate. For chicken's feeding or related prior tests, each chicken was an experimental unit and represented a replicate so that a mean for each feed source treatment was the average of seven chickens (*n* = 7) plus standard deviation.

## Results

### Employment of two T-DNA strategy to develop transgenic soybean

**Figure 1:**
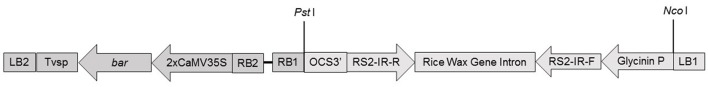
**Schematic show of 2 T-DNA regions of transformation construct pMU2T-bar-RS**. LB1 and RB1 as well as LB2 and RB2, T-DNA left border and right border regions 1 and 2, respectively; 2x CaMV35S, double CaMV35S promoter; *bar, b*i*a*laphos *r*esistance gene; Tvsp, soybean vegetative storage protein gene terminator; Glycinin P, soybean glycinin gene promoter; RS2-IR-F and RS2-IR-R, DNA forward and reverse inverted repeat sequences representing partial soybean *RS2* gene; OCS 3′, *Agrobacterium tumefaciens* Octopine gene terminator.

We employed two T-DNA construct pMU2T-bar-RS2 in which one T-DNA carried plant selectable marker while the other T-DNA contains RS2 silencing cassette (Figure 1). This allowed the independent integration of *bar* and RS2 transgene cassettes and thus subsequent segregation of the transgenes (Xing et al., 2000). Such marker-free plants are desirable for various subsequent utilities including commercial applications. RS2 cassette was driven by soybean glycinin gene promoter for seed-specific expression and rice-waxy gene intron separating the inverted repeats to ensure potent silencing. The inverted repeat was designed for a complete sequence homology with soybean *raffinose synthase 2* (*RS2*) gene but no significant homology with soybean *raffinose synthase 3* (*RS3*) gene (Figure S1). Using our soybean transformation protocol, we generated a total of 61 plants from an elite soybean genotype “Maverick.” Of those, 26 of 35 pMU2T-bar-RS2 events and 12 of 15 pMU2T-bar control events were herbicide resistant as shown by leaf painting (Figure S2).

PCR confirmed the presence of *bar* and RS2 transgenes of herbicide resistant events using two separate pairs of primers expanding partial regions of the two genes, respectively (Table S1). Because the two T-DNA regions within the vector could be separately integrated into the genome, some events would carry *bar* gene alone. Of 26 glufosinate-resistant pMU2T-bar-RS2 plants, 24 contained *bar*, of which only 9 also carried RS2 transgene, for a co-integration rate of 37.5%. Of 12 pMU2T-bar glufosinate resistant plants, all carried *bar* and were PCR-negative for RS2 transgene, as expected (data not shown).

### Progeny segregation analysis

T1 seedlings derived from the original nine T0 plants positive for both *bar* and RS2 transgenes were screened by leaf-painting and PCR for presence of *bar* and RS2 transgenes (Table S2). Of these nine lines, four were able to transmit both *bar* and RS2 transgenes to the progeny, for a co-inheritance rate of 44.4%. Because the *bar* and RS2 transgenes may integrate separately into the genome, T1 progeny analysis could reveal genetic linkage of the transgenes. However, some T0 events had higher rate of transgene inheritance than others, complicating the analysis. A Chi-square test was performed to test if the segregation fits normal 9:3:3:1 [bar(+)/RS2(+):bar(+)/RS2(−):bar(−)/RS2(+):bar(−)/RS2(−)] distribution to indicate an independent distribution of *bar* and RS2 transgenes (Table 1). Because Chi-square values for lines ZM3-7, ZM4-1, and ZM1-1 do not fit 9:3:3:1, and only seven bar(−)/RS2(+) plants were identified in 185 plants of all lines, we assumed that the transgene segregation for *bar* and *RS2* at the T1 generation does not follow Mendelian patterns.

**Table 1:** **T1 segregation of ***bar*** and RS2 transgenes and test of Goodness of Fit for 9:3:3:1 model**.

**T0 Event**	**Bar(+)/RS2(+)**	**Bar(+)/RS2(−)**	**Bar(−)/RS2(+)**	**Bar(−)/RS2(−)**	**Chi-square**	**Reject 9:3:3:1 model?**
ZM-1-1	2	3	0	15	162	Yes
ZM-3-7	3	5	6	3	11.6	Yes
ZM-4-1	6	1	1	5	23.5	Yes
MM-3-2	13	5	0	1	4.7	Yes

*Rejection of 9:3:3:1 model of independent segregation is at p = 0.05 and 3° of freedom*.

We further conducted T2 progeny segregation analysis to select a homozygous bar(−)/RS2(+) T1 line—carrying RS2 transgene but lacking the selectable marker—which is commercially valuable. Of the 17 T2 progeny leaf-painted from the T1 line MM3-2-15, none were resistant, and all 12 analyzed by PCR were positive for the RS2 transgene. The line MM3-2-10, which shared a T0 parent with MM3-2-15 was found to be resistant to glufosinate in 9/9 plants and RS2(−) for 4/4 plants. Based on these results and as confirmed in T3 analysis, MM3-2-15 and MM3-2-10 were chosen as the representative homozygous bar(−)/RS2(+) and bar(+)/RS2(−) lines, respectively, for subsequent experiments.

### Confirmation of transgene genomic integration

**Figure 2:**
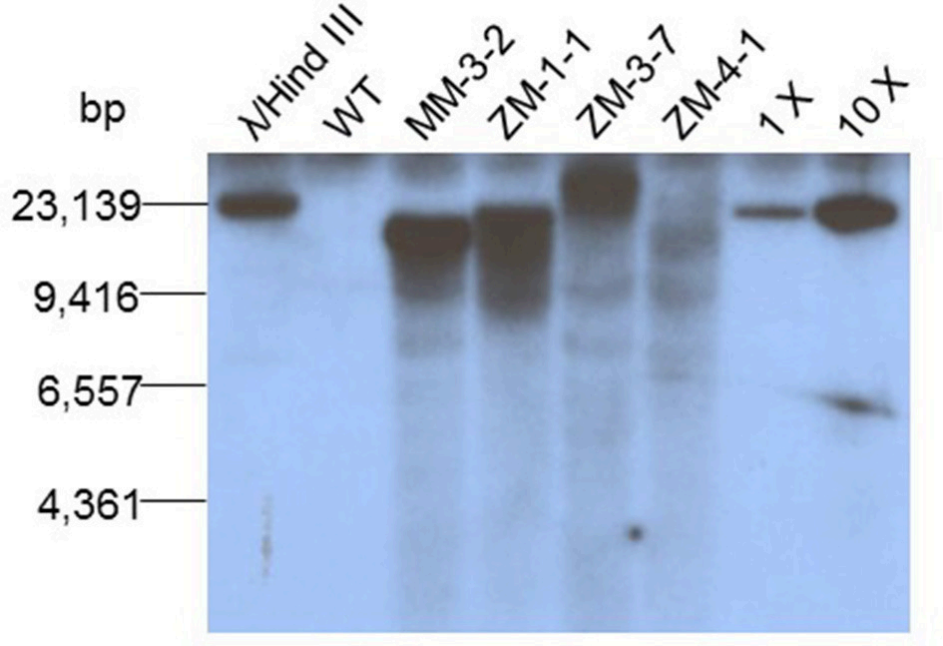
**Southern blot analysis of RS2 transgenic events**. From left to right: Lane 1, λ/*Hind* III DNA ladder; lane 2, wild type control; lanes 3–6, RS2 transgenic soybean events; lanes 7 and 8, 18 and 180 pg of construct pMU2T-bar-RS DNA as copy number control representing 1x and 10x genome equivalence, respectively.

We first performed leaf-painting and PCR screen to find out T1 plants that carried only RS2 transgene, i.e., bar(−)/RS2(+) genotype. A radiolabeled ^32^P Southern blot was done on genomic DNA to confirm RS2 transgene integration into the soybean genome. Four T1 plants representing their respective T0 events were analyzed which included four pMU2T-bar-RS2 transgenic events and one wild type negative control. The samples were digested with *Nco*I to cut once the first T-DNA region carrying RS2 cassette so that different events could be identified. Four T0 events carrying transgene RS2 were detected by the rice-waxy A gene intron (Figure 1) as a probe, confirming that events ZM1-1, MM3-2, ZM3-7, and ZM4-1 were indeed transgenic and independent and the gene was stably inherited to the T1 progeny (Figure 2). The wild type control sample didn't show any band as expected.

Two weak hybridization bands whose sizes were smaller than the RS2 transgene inserts appeared in nearly all transgenic samples. Our blast search in soybean genome sequence via Phytozome v.12.0 using the rice waxy intron sequence as a query detected two high homology regions on chromosomes 3 and 19, respectively. These two regions were located between 37825682–37826009 on chromosome 3 and 42570278–42570589 on chromosome 19, sharing 98.8 and 89.7% homology, respectively. These bands were nearly not detectable in wild type control sample presumably due to a lower amount of genomic DNA sample run in the gel. The weak bands might also result from a transgene-related background during genomic DNA preparation and digestion.

### Silencing of endogenous *RS2* mRNA

**Figure 3:**
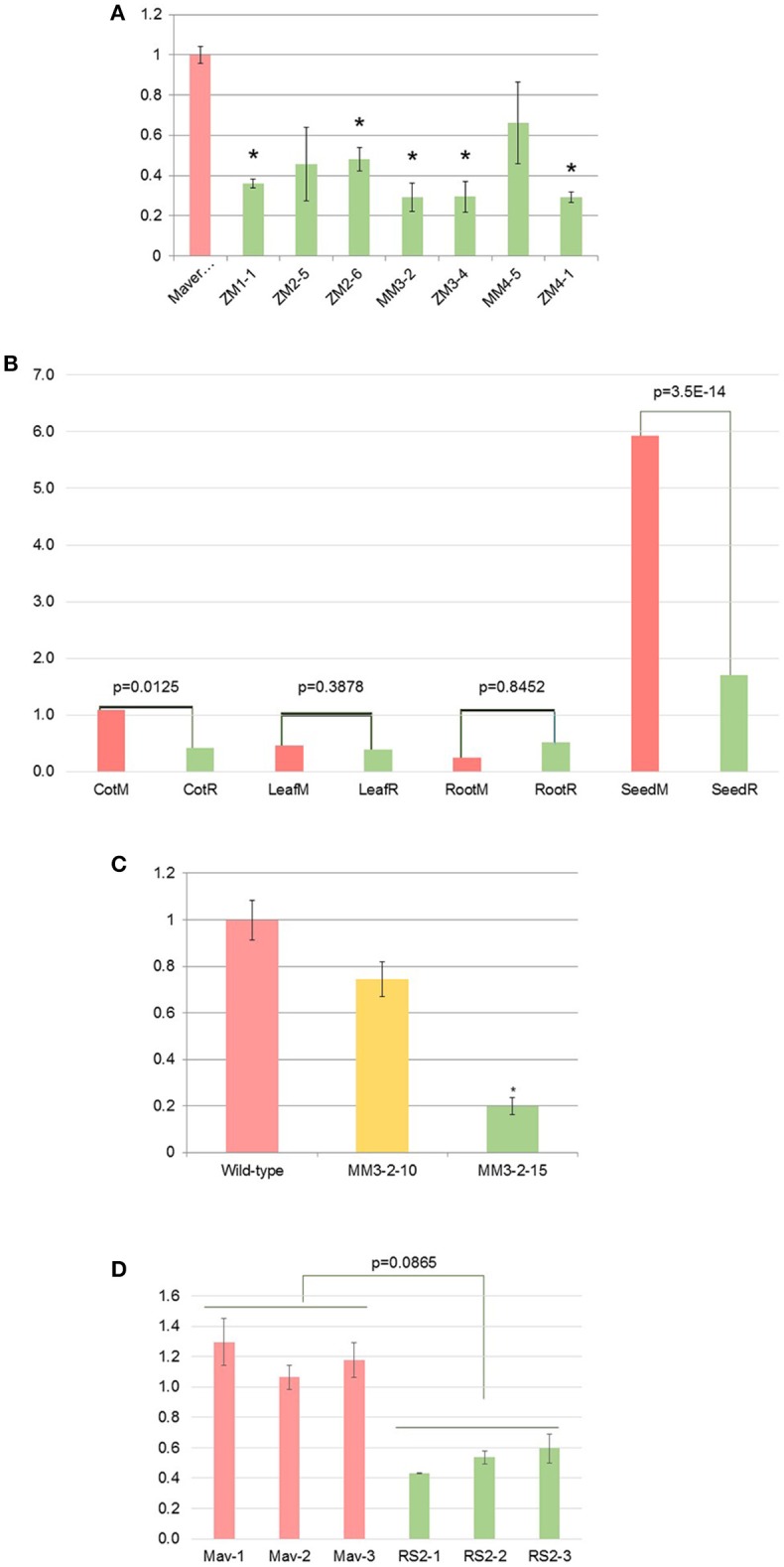
**Silencing of soybean endogenous ***RS2*** mRNA in T1 mid-mature seeds (A)**, various tissues: cotyledon, leaf, root, seed **(B)**, and T3 mid-mature seeds under the greenhouse **(C)** or field **(D)** conditions. M and R, Maverick (WT) and RS2 transgenic, respectively. X-axis represents wild-type (Maverick) as well as different RS2 transgenic events while Y-axis is the relative *RS2* mRNA level as determined by qRT-PCR and wild type is set to 1. Bar in each column means standard deviation. “^*^”Indicates statistical difference at *p* = 0.05 level.

T1 mid-mature seeds from six T0 plants were first confirmed to be transgenic by PCR screen, then mRNA levels of endogenous *RS2* were evaluated by qRT-PCR. The *RS2* mRNA in T1 seeds were suppressed 34% (MM4-5) to 71% (MM3-2 and ZM3-4) of the *RS2* mRNA in WT (Figure 3A). This magnitude of silencing in the mid-mature seeds was further examined spatially using various soybean plant issues, confirming that such a potent silencing was more seed-specific manner owing to the use of glycinin promoter (Figure 3B). Then, we identified homozygous lines MM3-2-15 (transgenic RS2 homozygous) and MM3-2-10 (*bar* homozygous) from T2 generation grown in the greenhouse. Mid-mature seeds were analyzed by qRT-PCR (Figure 3C). MM3-2-10 seeds showed 26% reduction of endogenous *RS2* mRNA (*p* = 0.084) whereas MM3-2-15 seeds had 80% reduction of *RS2* mRNA, a consistent downregulation with T1 seeds (71%). Under field conditions, endogenous *RS2* expression was 56% downregulated in transgenic MM3-2-15 compared to developing seeds (Figure 3D). The reduction in downregulation from greenhouse to field conditions could be due to environmental impacts of carbohydrate synthesis in developing seeds (Thomas et al., 2003).

We also examined the specificity of *RS2* inverted repeat to rule out its possibility of silencing *RS3* gene which shares 58.6% sequence identity with *RS2*. The RNA samples from field-grown mid-mature seeds were analyzed. No significant difference in *RS3* mRNA levels were observed compared to transgenic plants at *p* = 0.05 (Figure S3). This result also allowed for observed carbohydrate phenotypes to be attributed to the sole contribution and silencing of *RS2*.

### Carbohydrate profiles showed significant reductions of raffinose sugars in transgenic seeds

**Figure 4:**
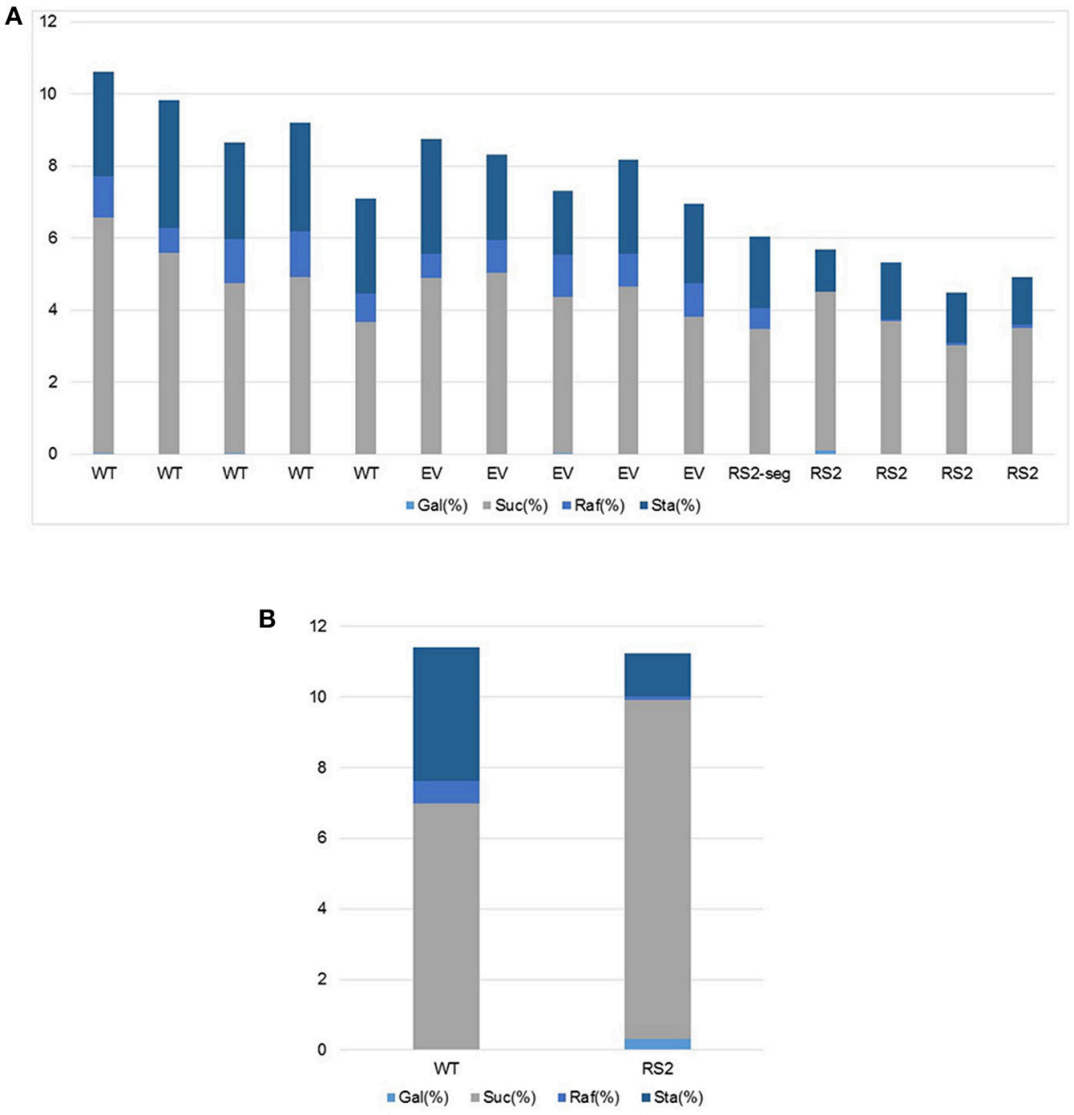
**Carbohydrate content in seeds from soybean plants grown under the greenhouse (A)** or field **(B)** conditions. X-axis represents wild-type (Maverick), empty vector (EV), or T3 RS2 transgenic events whereas Y-axis indicates percent (%) galactinol, sucrose, raffinose, and stachyose contents, respectively, in dry seeds of each individual plant assayed. *P*-value is provided when statistical significance is found.

Five individual seeds were analyzed from each of three lines, WT, empty vector control MM4-1, and RS2 transgenic MM3-2. High performance liquid chromatography (HPLC) analyzed relative contents of galactinol, sucrose, raffinose, and stachyose in mature soybean seeds. At this generation, line MM3-2 was still segregating for the transgene so one of the five seeds sampled had a carbohydrate profile similar to WT. Average values from each genotype are presented in Figure 4A. No significant difference in galactinol content was detected for the three lines. WT seeds had significantly more sucrose than RS2 transgenic, with average percent sucrose for WT, empty vector, and *RS2* transgenic being 5.08, 4.55, and 3.66, respectively. Raffinose content in WT and empty vector was significantly higher than RS2 transgenic at 1.03, 0.92, and 0.05%, respectively. Stachyose in WT and empty vector was also significantly higher than RS2 transgenic at 2.96, 2.43, and 1.37%, respectively.

To measure mature seed carbohydrate content more accurately and representative of field conditions, transgenic (MM3-2-15) and WT plants were grown side-by-side in a field plot (Figure S4). Fifteen T3 mature seeds from a bulk harvest of each genotype were analyzed with HPLC and Megazyme assay in two replications (Figure 4B). Galactinol in transgenic seeds was significantly higher than WT at 0.31 and 0.03%, respectively (*p* < 0.001). Sucrose was significantly increased in transgenic seeds compared to WT at 9.6 and 6.95%, respectively (*p* = 0.05). Both raffinose and stachyose contents were significantly reduced in transgenic seeds as compared to WT (raffinose *p* = 0.0015; stachyose *p* < 0.001). Raffinose content in transgenic seeds was 0.11%, compared with 0.63% in WT. Stachyose in transgenic seeds was 1.21%, and 3.79% in WT. Because the T3 experiment replicated real field conditions, and WT plants had identical growing conditions as transgenic, the T3 seed results are likely more representative of the *RS2* knock-down phenotype than T1.

### Composition analysis of field grown transgenic and WT seeds

Transgenic (MM3-2-15) and WT plants were grown side-by-side in a field plot to increase seeds. Ten randomly chosen plants for each WT and transgenic were leaf-painted for absence of *bar* gene and, as expected, all leaves were susceptible. Mid-mature seeds corresponding to roughly stage R5/R6 were analyzed for *RS2* and *RS3* gene expression by qRT-PCR. No visible difference in plant growth or maturation was observed throughout the growing season (Figure S4). Total seed yields for MM3-2-15 and WT were 6.44 and 5.77 kg. This result contradicts the yield reduction observed in greenhouse-grown plants. However, the field results are likely more representative of the true yield potential of transgenic, low-raffinose plants. To determine if the reduction in carbohydrate content affected seed fat, crude protein, or amino acid composition, samples of field-grown seed were analyzed. There were no substantial differences between the transgenic and WT lines in moisture content (6.13 and 6.14%, respectively), crude fat (19.18 and 19.79%, respectively), ash (5.21 and 5.27%, respectively), crude protein (37.26 and 36.14%, respectively), or for any of the amino acids. Crude fiber appeared reduced in transgenic (6.56%) compared to WT (7.07%). This result was similar to that by Chen et al. (2013), so it is possible that the alterations in sucrose and oligosaccharide also affected total fiber content.

### RS2 transgenic seeds increased true metabolizable energy in chicken feeding study

Prior to chicken feeding study, we first analyzed seed carbohydrate compositions (Table 2). While WT seeds had more total soluble carbohydrates than RS2 transgenic seeds, i.e., 11.4 and 10.9 g, respectively, the proportion of digestible carbohydrates was much higher for RS2 transgenic seeds with low-raffinose. The low-raffinose soybean had about half of raffinose, and <5% of stachyose of WT. Also, low-raffinose seed had about 20% more sucrose than WT. The major reduction in indigestible oligosaccharides and increase in sucrose are expected knowing the genotypes of these soybean varieties. The gross energy of the low-oligosaccharide soybean meal as measured by bomb calorimetry was slightly higher at 5371 kcal/kg than WT soybean meal at 5340 kcal/kg.

**Table 2:** Carbohydrate composition of soybean lines used in the study^*^.

**Soybean line**	**Galactinol**	**Sucrose**	**Raffinose**	**Stachyose**
RS2	0.31a	9.6a	0.11a	1.21a
WT	0.03b	6.95b	0.63b	3.79b

* *Carbohydrate composition is expressed as % seed weight*.

*The numbers followed by different letters indicate statistical difference at p = 0.05 level*.

**Figure 5:**
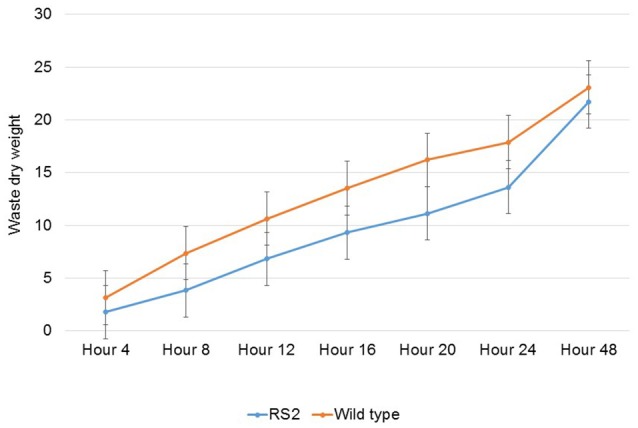
**Passage rate of feedstuff of wild type and transgenic soybean seeds**. X-axis means different time points for sampling from rooster wastes while Y-axis indicates percent (%) waste dry weight passed the gut as running total. RS2 and Mav, RS2 transgenic, and wild type Maverick seeds, respectively. Error bar indicates the standard error of a mean.

Then, we assayed the passage rate of feedstuff. To do this, samples were collected every 4 h for the first 24 h after feeding because there has been evidence that oligosaccharide content in poultry diets influences passage rate (Coon et al., 1990). The results showed that the passage rate of the low-raffinose diet was a little slower than WT soybean meal (Figure 5). Moreover, we analyzed dry matter digestibility of soybean seeds. To this end, we first calculated endogenous loss to correct for the amount of endogenous urine and fecal energy lost during the 48 h period test. Total endogenous loss for the entire period averaged 3.48 g of dry excreta per chicken for all chickens in the study. The average endogenous loss value was subtracted from the after-feeding excreta weight for each chicken.

Dry matter digestibility was then calculated by subtracting the dry weight of excreta from the weight of feed, which was 30 g per chicken, and then dividing that by the weight of feed Table 3A). The dry matter digestibility for WT soybean meal was 23.1%, compared with 27.6% for low-raffinose soybean meal (Table 3B). Finally and most importantly, we measured true metabolizable energy using calculation in Sibbald (1976). The TME for WT soybean meal was 2385 kcal/kg, which is consistent with the usual TME of soybean meal (Table 4). The TME for low-raffinose soybean meal was 2734 kcal/kg, for a 14.6% increase (Table 4). This increase in TME shows that low-raffinose soybean meal could have a positive impact on the animal health and profitability of animal feeding operations.

**Table 3A:** **Feed calculations (***n*** = 2)**.

	**GE of feed (cal/g)**	**Weight of feed (g)**	**Kcal in feed per chicken**
RS2	5371 ± 152.62	30	161.12 ± 4.59
WT	5340 ± 210.59	30	160.20 ± 6.32

**Table 3B:** **Excreta calculations (***n*** = 7)**.

	**GE of excreta (cal/g)**	**Weight of excreta (g)**	**Dry matter digestibility (%)**	**Endogenous loss (g)**	**Kcal in waste per chicken**
RS2	4321.55 ± 545.98	21.72 ± 1.82	27.6	3.48	79.10 ± 14.69
WT	4513.67 ± 213.39	23.07 ± 2.21	23.1		88.64 ± 12.75
	*p* = 0.4029	*p* = 0.2355			*p* = 0.2188

*GE means gross energy*.

**Table 4:** **True metabolic energy (TME) calculations (***n*** = 7)**.

	**Kcal absorbed by chicken from 30 g feed**	**TME (kcal/kg)**
RS2	82.027 ± 14.69	2734.25 ± 489.70
WT	71.564 ± 12.75	2385.46 ± 424.99
		*p* = 0.1802

## Discussion

We have achieved increased true metabolizable energy of poultry feed in soybean seeds by silencing soybean *RS2* through RNAi. In the current work, results for transgene co-integration, transgene inheritance, T1 seedling vigor, carbohydrate phenotype, and yield were somewhat unexpected and deserve additional examination. Of the 24 *bar*-positive events generated from transformation using the pMU2T-bar-RS2 plasmid, nine also had the RS2 transgene of interest. This 37.5% co-integration rate is lower than the 47% rate reported for rice and tobacco in Komari et al. (1996) and the 70% rate for soybean (Xing et al., 2000). Of the nine co-integration events, four passed both transgenes to the progeny, and marker-free progeny were observed in all four lines by T3 generation. Thus, 4 out of 24 events, or 16.6% produced transgene-positive, marker-free progeny. Interestingly, Matthews et al. (2001) also reported 16% of transformed barley events producing transgene-positive, marker-free progeny using a “twin T-DNA” vector where the two T-DNA borders are immediately adjacent to one another. This information is useful to future researchers wishing to obtain marker-free transgenic events, as it shows that about one in six events produced will yield progeny with the desired genotype using the pMU2T-bar plasmid.

It has been well-established that seed quality affects seedling vigor and growth (Abdul-Baki and Anderson, 1973; Parrish and Leopold, 1978), and factors such as temperature, genotype, humidity, and fungal contamination contribute to seed quality (TeKrony et al., 1984; Dornbos et al., 1989; Gibson and Mullen, 1996; Egli et al., 2005). Greenhouse conditions during summer months when the T0 plants were growing were hot and humid, which likely contributed to the poor seed quality and seedling vigor observed for T1 seedlings. Although plant growth was stunted to some degree in T1 transgenic plants, no obvious stunting was noted in the T2 or T3 generations, thus seed quality and seedling vigor are assumed to be dependent on external factors or effects of tissue culture rather than the transgene.

For the T1 carbohydrate contents (Figure 4A), the decrease of sucrose in RS2 transgenic seeds is unexpected given the known raffinose biosynthesis pathway. This result could be due to effects of tissue culture on the T0 plant or different growing conditions of transgenic and WT plants. WT plants were sown in the greenhouse to be used as a control at about the same time as transgenic plants were transferred to the greenhouse, but transgenic plants often flowered sooner than their WT counterparts. The strong environmental influence on carbohydrate partitioning in developing seeds likely contributed much of the variability in sucrose content. To elucidate the effect of only transgene on soybean carbohydrate content, samples were obtained from field-grown plants with WT controls planted at the same time under the same field conditions. This comparative result showed a relative increase in sucrose content in RS2 transgenic (Figure 4B). Thus, the field results are likely more indicative of the true low-raffinose, high-sucrose phenotype expected in transgenic plants. Nevertheless, further studies are warranted.

This work led to a low-raffinose line of marker-free transgenic soybean. For commercial transgenic crops, yield parity is one of the most important traits, and though total seed yield was significantly reduced in the greenhouse yield study, yield was comparable between WT and transgenic plants under the field conditions. These lines are likely not commercial products, but this work is separate confirmation of the contribution of RS2 gene to raffinose accumulation in soybean seeds. The lines described in Bilyeu and Wiebold (2016) were from non-elite lines, and it would require several generations of backcrossing to obtain yield parity with modern commercial lines. The transformation approach illustrated in this present study provides a proof that low raffinose family could be obtained in elite soybean variety by transformation. Future experiments are needed to evaluate the inheritance of transgenes through several more generations, as well as the carbohydrate phenotype over generations and environmental conditions. Also, replicated, controlled experiments are needed to precisely measure agronomic traits such as emergence, stress tolerance, maturation, and yield for the transgenic plants. The line MM3-2-15 is certainly a strong candidate for use in improving soybean carbohydrate quality.

The result from chicken feeding study is encouraging which provides a baseline for the use of full-fat, reduced-oligosaccharide soybean meal in chicken diets. Further, feeding test would be desired using a larger number of chickens to reduce chicken-to-chicken variations (standard deviations), rendering TME between RS2 and WT statistical significant. In addition, more experiments should be conducted to better measure the contribution of only carbohydrate profile on true metabolizable energy using soybeans grown side-by-side in the field during a growing season. Oligosaccharide content and overall low metabolizable energy is a deterrent of using high percentages of soybean meal in poultry diets, but the use of genetically improved, low-oligosaccharide soy as a whole-bean protein, oil, and carbohydrate source may increase metabolizable energy in the diet while maintaining a low formulation cost.

## Author contributions

ZZ conceived and supervised the study; MV, JF, and ZZ designed the study; MV, JD, and MM performed the experiments; MV analyzed results and wrote manuscript; JD, MM, and ZZ revised manuscript; JF provided experimental materials; all approved manuscript.

### Conflict of interest statement

The authors declare that the research was conducted in the absence of any commercial or financial relationships that could be construed as a potential conflict of interest.
