# Supplementary material for: Silencing of Soybean Raffinose Synthase Gene Reduced Raffinose Family Oligosaccharides and Increased True Metabolizable Energy of Poultry Feed
Source: Front Plant Sci. 2017 May 16;8:692. doi: 10.3389/fpls.2017.00692 (PMC5432567; doi:10.3389/fpls.2017.00692)
Supplement: Supplementary file 1 [file DataSheet1.docx]

**Supplemental Figure Legends:**

**Figure S1** Sequence alignments between 300 base-pair RS2 inverted repeat (RS2_IR) and soybean raffinose synthase genes (**A**) *RS2* (Glyma.06G179200.1) and (**B**) *RS3* (Glyma.05G003900.1) using ClustalW (<http://www.genome.jp/tools/clustalw/)>. The inverted repeat sequence is an exact match for nucleotide from 580 to 879 of *RS2* genomic sequence, and nucleotide 97 to 396 of the coding sequence. Although the entire sequences of RS2 and RS3 have a high degree of identity (64%), the region of the inverted repeat sequence has low identity between RS2 and RS3 (58.6%) allowing more specific down-regulation of only the RS2 endogenous gene. (**C**) Genomic structure of the soybean *RS2* gene. Location of the 300bp RS2 inverted repeat is shown in blue in exon 1.

**Figure S2** Representative result of leaf paint using 100mg/L glufosinate, showing (**A**) wild-type susceptible and (**B**) transgenic resistant 3 days after herbicide application. Susceptible leaves will eventually become necrotic and die.

**Figure S3** Silencing of soybean endogenous *RS3* mRNA in T1 mid-mature seeds. M and R, Maverick (WT) and RS2 transgenic, respectively. X-axis represents wild-type (Maverick) as well as different *RS2* transgenic events while Y-axis is the relative *RS3* mRNA level as determined by qRT-PCR and wild type is set to 1. Bar in each column means standard deviation and represents a 10-seed subsample of pooled mid-mature seeds.

**Figure S4** Field plot of wild type and RS2 transgenic soybean production. (**A**) planting, (**B**) young seedlings, (**C**) vegetative growth prior to flowering, and (**D**) ready for seed harvest. Four rows of 70 seeds for each transgenic and wild-type seeds were protected by open zones.

| **Table S1** Primers used for various PCR amplifications | |  |
| --- | --- | --- |
|  |  |  |
| Primer name | Primer sequences 5' to 3' | Description |
|  |  |  |
| R T-DNA F1 | CTGCTGCAGAAT CTCGAGCA | Forward primer for first T-DNA right border |
|  | CTGGCCGTCGTTTTACAAC |  |
| R T-DNA R1 | GCGCATGCGACGTCATTTAAATTG | Reverse primer for first T-DNA right border |
|  | ACAGGATATATTGGCGGGTAAAC |  |
|  | CAAATGGACGAACGGATAAACC |  |
| L T-DNA F2 | GCGGACGTCGGCGCGCCACTAGTGCAC | Forward primer for second T-DNA left border |
|  | CATGGAGGCGGTTTGCGTATTGGCTAG |  |
| L T-DNA R2 | GCAGCATGCCGAGTGGTGATTT TGTGCCGAGC | Reverse primer for second T-DNA left border |
| bar-ORF-sense | CACCATCGTCAACCACTACATCG | Sense primer for detection of bar gene |
| bar-ORF-antisense | CAGCAGGTGGGTGTAGAGCGT | Antisense primer for detection of bar gene |
| Rice waxy-intron reverse | GTCATATCCCCTAGCCACCC | Reverse primer for detection of RS2 transgene |
| OCS 3’ forward | TCATGCGATCATAGGCGTCT | Forward primer for detection of RS2 transgene |
| CaMV-Right | GAAACCTCCTCGGATTCCAT | Foreward primer for contamination-free detection of bar gene |
| bar ORF reverse | CAGCAGGTGGGTGTAGAGCGT | Reverse primer for contamination-free detection of bar gene |
| 1a-fwd | GACCTTCTTCGTTTCTCGCA | Forward primer for qRT-PCR for Elongation Factor 1a control |
| 1a-rvs | CGAACCTCTCAATCACACGC | Reverse primer for qRT-PCR for Elongation Factor 1a control |
| RS2 fwd | CTAGGGCCATCTCTGGTGGA | Forward primer for qRT-PCR for RS2 endogenous gene |
| RS2 rvs | CGTGTGGGGAGTGCATAGTG | Reverse primer for qRT-PCR for RS2 endogenous gene |
| RS3 fwd | CACTGGAGTTCTTGGGGTGT | Forward primer for qRT-PCR for RS3 endogenous gene |
| RS3 rvs | GCTTGGCTGAAATACGAAGC | Reverse primer for qRT-PCR for RS3 endogenous gene |
|  |  |  |

**Table S2** Summary of T1 genotype, *raffinose synthase 2* (*RS2*) transcript levels, and progeny segregation

|  |  |  |  |  |  |  |  |
| --- | --- | --- | --- | --- | --- | --- | --- |
| T0 event | Leaf-paint | PCR bar | PRC RS2 | qRT-PCR** | No. progeny | Progeny bar (+) | Progeny RS (+) |
|  |  |  |  |  |  |  |  |
|  |  |  |  |  |  |  |  |
| ZM-1-1* | RRR | Yes | Yes | 64% | 22 | 5 | 2 |
| ZM-2-5 | RRR | Yes | Yes | 54% | 30 | 0 | 0 |
| ZM-2-6 | RRR | Yes | Yes | 52% | 27 | 0 | 0 |
| ZM-3-4 | RRR | Yes | Yes | 71% | 17 | 0 | 0 |
| ZM-3-7* | RRR | Yes | Yes | ND | 20 | 8 | 9 |
| ZM-4-1* | RRR | Yes | Yes | 71% | 14 | 7 | 7 |
| MM-2-4 | RRR | Yes | Yes | ND | 19 | 0 | 0 |
| MM-3-2* | RRR | Yes | Yes | 71% | 19 | 18 | 13 |
| MM-4-5 | RRR | Yes | Yes | 34% | 17 | 0 | 0 |
|  |  |  |  |  |  |  |  |

*: Events transmitted transgene to the progeny; **: % down-regulation; ND: not determined due to insufficient T1 seeds

RRR: Leaf paint results of three times showing resistance to herbicide glufosinate (100mg/L)
