# Supplementary figures and images for: Silencing of Soybean Raffinose Synthase Gene Reduced Raffinose Family Oligosaccharides and Increased True Metabolizable Energy of Poultry Feed
Source: Front Plant Sci. 2017 May 16;8:692. doi: 10.3389/fpls.2017.00692 (PMC5432567; doi:10.3389/fpls.2017.00692)

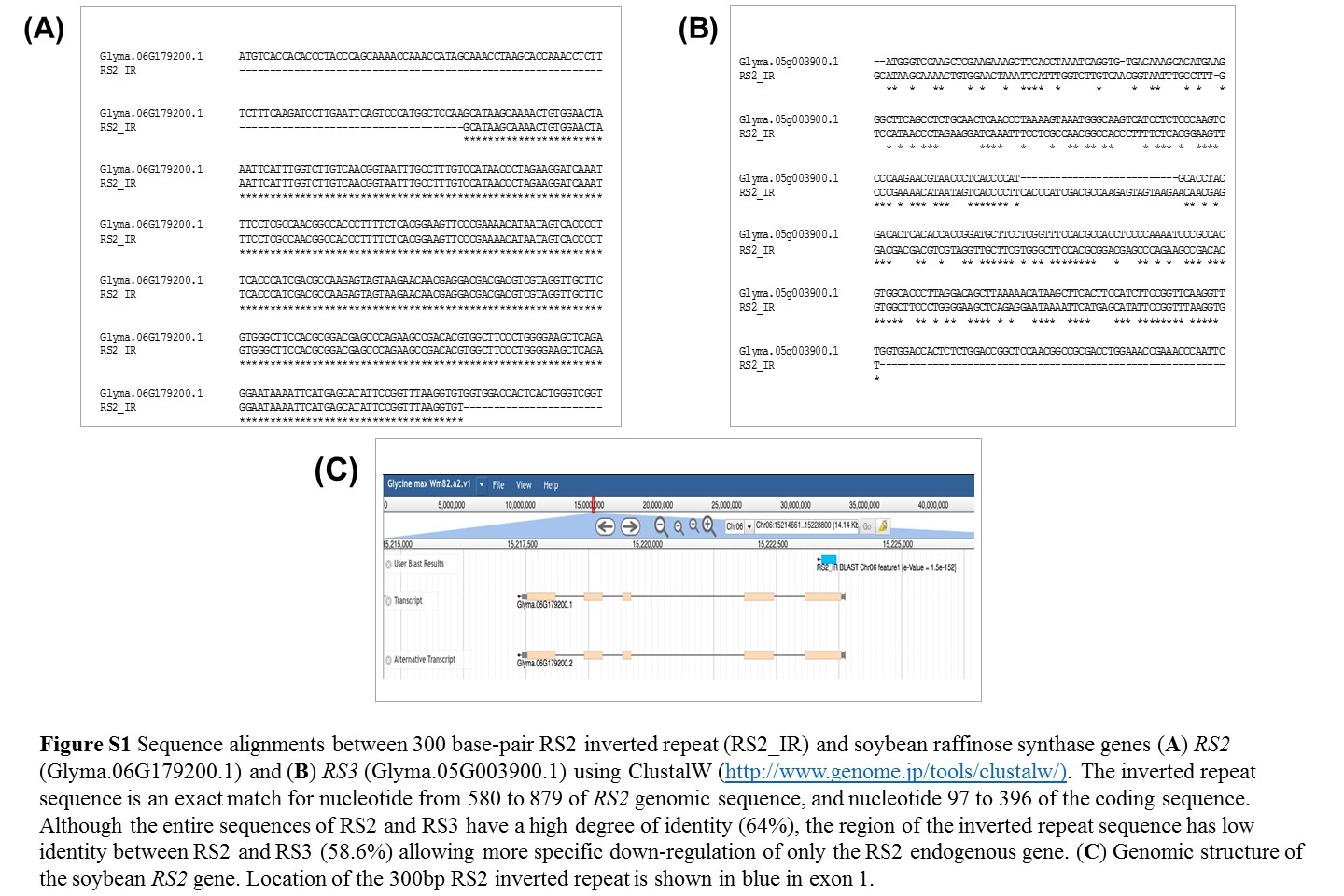

Supplement: Supplementary file 2 [file Image1.JPEG]

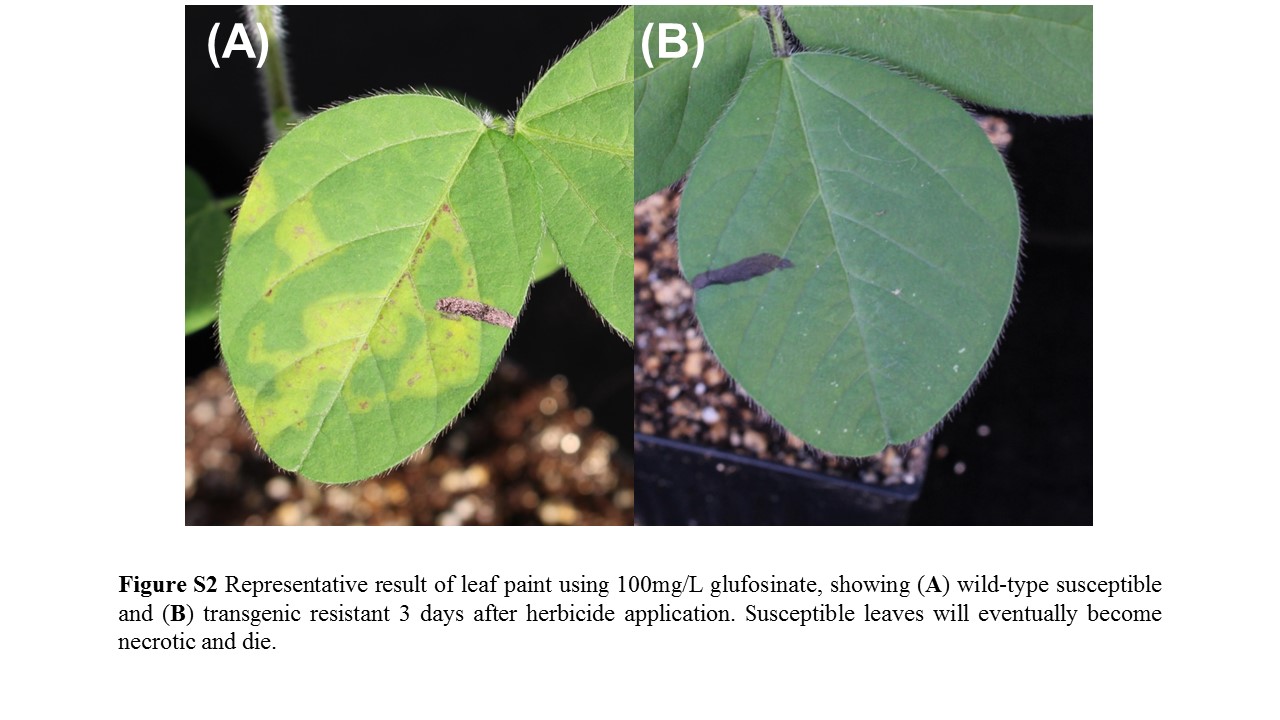

Supplement: Supplementary file 3 [file Image2.JPEG]

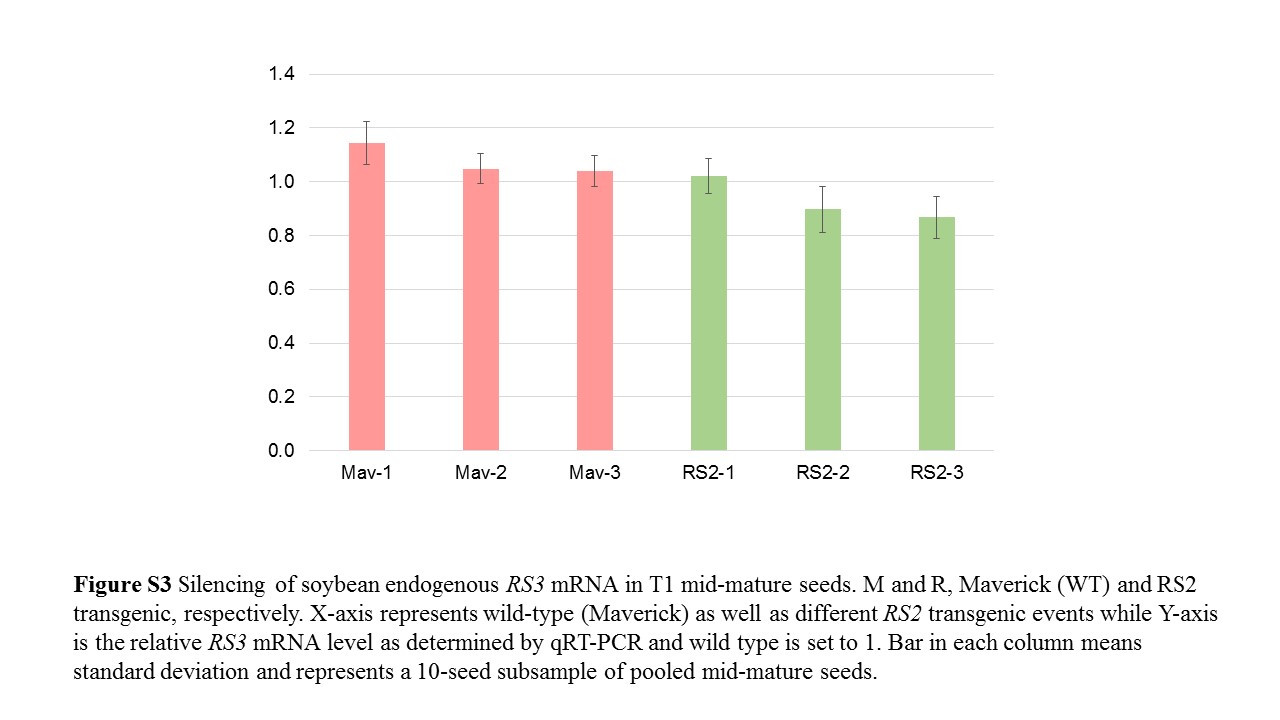

Supplement: Supplementary file 4 [file Image3.JPEG]

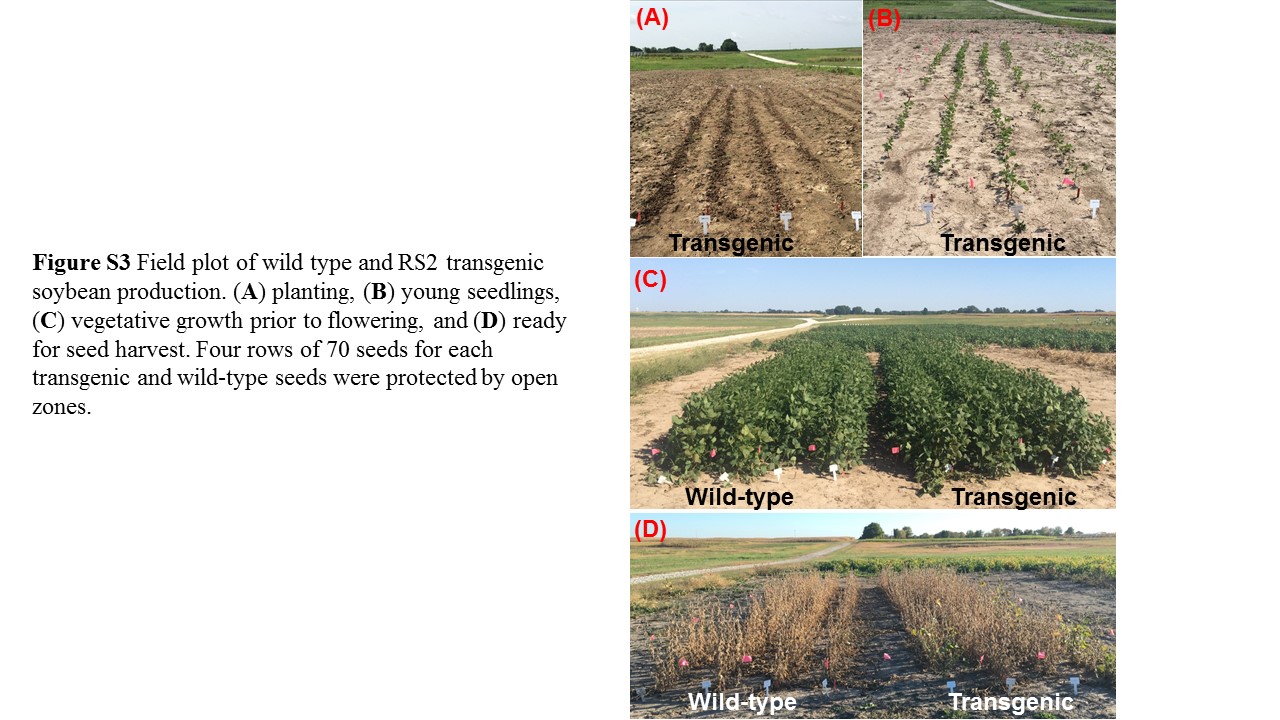

Supplement: Supplementary file 5 [file Image4.JPEG]
